# Insights from semi-structured interviews on integrating artificial intelligence in clinical chemistry laboratory practices

**DOI:** 10.1186/s12909-024-05078-x

**Published:** 2024-02-22

**Authors:** Lena Jafri, Arsala Jameel Farooqui, Janet Grant, Usmaan Omer, Rodney Gale, Sibtain Ahmed, Aysha Habib Khan, Imran Siddiqui, Farooq Ghani, Hafsa Majid

**Affiliations:** 1 Section of Chemical Pathology, Department of Pathology and Laboratory Medicine, Aga Khan University, 74800 Karachi, Pakistan; 2 Centre for Medical Education in Context [CenMEDIC], CenMEDIC, 27 Church Street, TW12 2EB Hampton, Middlesex UK; 3 Putnam PHMR, NE4 5TF Newcastle, UK

**Keywords:** Artificial intelligence, Interviews, Change management, Medical education, Clinical Chemistry, Laboratory

## Abstract

**Background:**

Artificial intelligence (AI) is gradually transforming the practises of healthcare providers. Over the last two decades, the advent of AI into numerous aspects of pathology has opened transformative possibilities in how we practise laboratory medicine. Objectives of this study were to explore how AI could impact the clinical practices of professionals working in Clinical Chemistry laboratories, while also identifying effective strategies in medical education to facilitate the required changes.

**Methods:**

From March to August 2022, an exploratory qualitative study was conducted at the Section of Clinical Chemistry, Department of Pathology and Laboratory Medicine, Aga Khan University, Karachi, Pakistan, in collaboration with Keele University, Newcastle, United Kingdom. Semi-structured interviews were conducted to collect information from diverse group of professionals working in Clinical Chemistry laboratories. All interviews were audio recorded and transcribed verbatim. They were asked what changes AI would involve in the laboratory, what resources would be necessary, and how medical education would assist them in adapting to the change. A content analysis was conducted, resulting in the development of codes and themes based on the analyzed data.

**Results:**

The interviews were analysed to identify three primary themes: perspectives and considerations for AI adoption, educational and curriculum adjustments, and implementation techniques. Although the use of diagnostic algorithms is currently limited in Pakistani Clinical Chemistry laboratories, the application of AI is expanding. All thirteen participants stated their reasons for being hesitant to use AI. Participants stressed the importance of critical aspects for effective AI deployment, the need of a collaborative integrative approach, and the need for constant horizon scanning to keep up with AI developments.

**Conclusions:**

Three primary themes related to AI adoption were identified: perspectives and considerations, educational and curriculum adjustments, and implementation techniques. The study’s findings give a sound foundation for making suggestions to clinical laboratories, scientific bodies, and national and international Clinical Chemistry and laboratory medicine organisations on how to manage pathologists’ shifting practises because of AI.

## Background

Disruptive innovation can be described as an invention that disrupts an existing market by introducing a novel concept or technology that offers an alternative set of values [1]. Given the heavy reliance on technology in pathology and laboratory medicine, these fields present a fertile ground for the emergence of disruptive breakthroughs [2, 3]. Throughout the history of laboratory advancements, disruptive innovation has demonstrated a pattern of initially slow growth, eventually gaining momentum and significantly displacing established standards [4]. Clinical Chemistry, also referred to as Chemical Pathology or Clinical Biochemistry, has witnessed significant adaptation to various disruptive innovations in recent decades. These innovations encompass total laboratory automation (TLA), point-of-care testing, mass spectrometry-based omics, and the detection of disease markers in liquid biopsy etc. [5–7, 8]. Initially implemented on an ad hoc basis, these disruptive innovations in Clinical Chemistry gradually became an integral part of routine practice [9].

A recent disruptive innovation is the field of artificial intelligence (AI) [2, 10]. AI is a science that employs computers to simulate human intellect with minimal human intervention [11]. Researchers in the fields of AI and biomedical informatics have raised concerns regarding the potential impact of technology on the medical workforce [12]. Although some experts remain sceptical about a technological revolution in healthcare, many healthcare providers recognize the significant potential of AI advancements to revolutionize the administration of healthcare [13–15].

What impact can disruptive advancements like AI have on pathologists working in Clinical Chemistry? The rapid pace and disruptive nature of AI can present challenges in terms of training and adapting to the changes it brings [16–18]. In the current landscape of advancing AI technologies in heathcare, it’s important for laboratory staff to consistently improve their knowledge and skills and stay open to disruptive innovations [19]. To achieve this goal, medical education plays a crucial role in comprehending learner motivations, transforming experiences, and delineating responsibilities. Considering the ongoing debate and uncertainties surrounding the influence of AI on the future of Clinical Chemistry, the research questions were as follows: (1) Addressing changes in practice management due to AI in Clinical Chemistry laboratories, (2) Identifying essential skills and support for smooth adaptation to AI in clinical Chemistry, and (3) Determining the necessary level of medical education intervention for effective integration of AI-induced practice changes. The objectives of this study were to investigate the impact of AI on Clinical Chemistry laboratories, to analyze strategies for effectively managing practice changes, identify essential skills for seamless adaptation within the realm of Clinical Chemistry, and ascertain the critical level of medical education intervention needed to facilitate a successful integration of AI in the field. This was achieved by evaluating the responses obtained from multisite semi-structured interviews conducted with a diverse group of professionals in the field of Clinical Chemistry in Pakistan.

## Methods

### Study design and setting

A collaborative exploratory qualitative investigation took place from March to August 2022 at the Section of Clinical Chemistry, Department of Pathology and Laboratory Medicine, Aga Khan University (AKU), Karachi Pakistan, in conjunction with Keele University, Newcastle United Kingdom. While AKU served as the primary data collection site, Keele University played a pivotal role along with principal investigator (PI) in the study’s conceptualization, methodology design, planning, and training of PI in qualitative research and semi-structured interview techniques.

### Study participants

Through purposeful sampling, professionals from Clinical Chemistry, including individuals at different career stages (junior, mid-level, and senior), were interviewed at various teaching hospitals in Pakistan. Informed consent was obtained from each participant prior to the interviews. The qualified individuals in Pakistan who were eligible to participate included consultant pathologists, whether working in public or private Clinical Chemistry laboratories, who had successfully completed the fellowship exit examination and were registered with the College of Physicians and Surgeons of Pakistan (CPSP). This also encompassed laboratory managers and technologists practicing in Clinical Chemistry laboratories affiliated with hospitals in Pakistan, as well as postgraduate trainees of Clinical Chemistry who were registered with CPSP.

### Data collection

The interview schedule was designed following an extensive review of the relevant literature. The interview schedule comprised the following inquiries:

- What implications and preparations does AI necessitate in healthcare, and how can education equip healthcare providers for these changes?
- How will clinical laboratories handle practice changes resulting from disruptive technologies like AI?
- What skills and support are essential for a seamless adaptation to the current scenario involving AI in Clinical Chemistry?
- At what stage of medical education should initiatives be taken to implement changes in practice?

The interview agenda included prompts to direct the conversation with essential subjects; a guide to provide structure and focus; and probes and clarification questions to go deeper and deepen our understanding. These elements combined to establish a strong framework for effective information gathering from participants and analysis.

Prior to the main study, a pilot study was conducted with two participants to assess the effectiveness of the interview schedule and to refine its language and style. Both pilot interviews were recorded, transcribed, and analysed. The findings of the pilot study revealed that the interview questions were thorough and generated detailed responses that addressed the research questions. As a result, a sample size of > 10 participants were determined to be appropriate for the main study based on the results of the pilot study.

Participants were enrolled via an email containing an information sheet that outlined their roles, the intended use of research data, and consent forms. At the beginning of each interview, participants were provided with a briefing on the study’s title and objectives. The interviews were recorded using a dictaphone or Zoom. The semi-structured interview schedule consisted of open-ended questions and prompts, derived from the existing literature, to ensure alignment with the research objectives [17]. The questions were formulated in clear and unambiguous English, prioritizing their clarity [18].

The recorded interviews were transcribed verbatim. Following the transcription, the data underwent coding to ensure confidentiality, and any identifying information was subsequently removed. The transcripts, audio files, consent forms, and notes were securely stored and labelled with pseudonyms to protect confidentiality. The principal investigator maintained a reflective diary to document personal thoughts on the data gathered during interviews, and before reading the transcripts, listened to the audio recordings and took notes. Thorne’s (2000) approach was followed to minimize personal or researcher bias towards the research questions.

### Data analysis and validation

The data gathered was analyzed using content analysis through annotation and theorization of meanings [20]. The interview transcripts were analyzed by breaking down each statement into smaller units of text and assigning codes based on their meaning or subject matter. Each interview was coded line by line, with a focus on identifying recurring patterns and key concepts. These codes were grouped together to create higher-level categories, and similar codes were merged to create overarching themes that extracted the key issues from the data in a comprehensive and interpretive manner. The themes were derived from the data itself to avoid relying solely on interview questions. After coding the interview scripts, a research associate reviewed them and made some changes and categorizations. The associate noticed that in some cases, the PI had assigned one unit to multiple codes, which is called double or simultaneous coding. Both coders underwent comprehensive discussions to familiarize themselves with the coding criteria and methodology. Regular meetings were organized to discuss coding discrepancies and clarify any ambiguities in the coding process. This iterative feedback loop allowed us to refine the coding guidelines and enhance coder agreement. To further ensure intercoder agreement, the research associate and PI analysed the recurring themes together and reached a point of 100% agreement on the coded data. The data is presented using carefully selected illustrative quotes that accurately capture the diverse perspectives discussed in the interviews, obtained with informed written consent from the participants. To attain validation, we conducted a comprehensive analysis of the data, collaborating with two colleagues to gain additional perspectives. They conducted additional validity readings to minimize subjectivity in the analyses and extracted a dependable and widely applicable set of factors from the interviews, which were deemed crucial in managing the practice change of pathologists prompted by AI. Validation was strengthened through a review of the initial analysis draft by supervisors at Keele University.

### Data statement

The data is presented in this study utilizing carefully selected illustrative quotes that accurately represent the arguments presented during the interviews and reflect the diverse range of perspectives expressed. During the selection of quotes, consideration was also given to their comprehensibility outside of their original context. It is crucial to note that the complete datasets are not publicly accessible to ensure the privacy protection of the participants.

## Results

In the sample of thirteen interviewees, gender distribution comprised five males and eight females, denoted as P1 through P13 for reference. The cohort included seven practicing pathologists, four postgraduate trainees, and one individual serving in the capacity of a laboratory manager and technologist. Senior (*n* = 5), mid-level (*n* = 4), and junior (*n* = 4) career phases were represented. Additionally, participants were drawn from both the public (20%) and private sectors (80%), providing a breadth of perspectives. The emergent topics are further developed under the subheadings below. The analysis of the interview data revealed three prominent themes that shed light on the perceptions and considerations for AI adoption, the necessary educational and curriculum adjustments, and the implementation techniques. Interview analysis revealed sub-themes addressing hesitations in AI adoption, potential benefits, curriculum changes, strategies, critical factors, collaborative integrative approach, and horizon scanning for AI advancements.

### Theme I: perceptions and considerations for AI integration

In the realm of Clinical Chemistry, the perception of integrating AI in practice is met with challenges, according to the responses from participants, ranging from concerns about job loss to a hesitancy to embrace change. Participants underscored the synergy between machines and human expertise, foreseeing higher-quality reports, faster diagnoses, and improved patient management through collaborative efforts.

#### Reasons for hesitation in adaptation of AI in clinical chemistry

Participants agreed that AI intimidates employees in Clinical Chemistry laboratory. They all shared the idea that the adoption of AI would result in job losses, which would be a significant hindrance to its implementation. Another impediment to AI application was the lack of acceptance of AI as a tool for improving clinical laboratory operations. P3, P4, P6, and P8 all addressed practical and scientific problems that laboratories encounter that impede the development of AI algorithms during the interviews. Three participants (P3, P6, P10) presented their previous difficult experiences in their laboratories with AI-algorithm-powered projects. They stressed the importance of properly validating diagnostic algorithms employing AI in the Pakistani population. All participants noticed a deficiency.

***P11:****“No one has ever taught us AI as such. Although now as we upgrade our lab, we can try that we bring this in our lab to elevate ourselves one step further.”*

Approximately half of the interviewees were unaware of the usage of AI in Pakistani laboratories. When questioned about the support needed to integrate AI into Clinical Chemistry laboratories, participants cited the inadequacy of modern AI technologies and supporting infrastructure as a major issue. Furthermore, one person raised concern about over-reliance on automated systems.

***P3:****“I don’t think that AI can build up their critical thinking abilities”.*

Another issue raised was the lack of standardisation in the quality of data generated by clinical laboratories across Pakistan. Data quality and sharing were questionable in the context of respondents’ concerns, and data utilisation was hampered by poor quality clinical laboratory data and a lack of standardisation among Clinical Chemistry laboratories.

Respondents emphasized that the application of AI in data interpretation has the potential to improve patient care. However, they stressed the importance of ensuring the accuracy and reliability of data to avoid any potential negative impacts or adverse consequences. According to some participants (*n* = 3), the absence of Logical Observation Identifiers Names and Coding (LOINC) in clinical laboratories impedes AI application. Discussions focused on the importance of LOINC codes in data integration and clinical decision-making. Implementing LOINC in these labs would help to overcome obstacles, boost AI integration, and eventually improve healthcare outcomes. One of the participants emphasized the need to establish AI principles in our country’s healthcare system, ensuring that not only the data can be readily accessible and reusable, but also the software and AI algorithms used in conjunction with the data are well-defined and standardized.

#### Embracing AI’s potential AI in clinical chemistry

The participants discussed potential areas where AI could be implemented within laboratory settings, and their insights have been summarized in the Table 1. They all recognised the potential of AI technologies to assist pathologists with the time-consuming and repetitive duties that pathologists face. They recognised AI’s potential to improve diagnostic accuracy by assisting pre-analytical, analytical, and post-analytical laboratory operations. AI integration in laboratory operations would improve efficiency, eliminate human error, and maximise resource utilisation. Participants also stressed the significance of collaboration between machines and human expertise, which leads to higher-quality laboratory reports, faster diagnosis, and better patient management. The interviews also highlighted that AI has the potential to aid with laboratory administration and operations, resulting in less need for laboratory managers to be available 24 h a day, seven days a week.

***P5:****“AI will help us in lessen the burden of work on a pathologist and a technologist and they can do specialized work only.”*

They emphasised the potential of AI and quality control tools to improve laboratory test report interpretation and assist pathologists in making more accurate diagnoses and judgements. They also stressed the significance of collaboration between Clinical Chemistry experts and AI experts from other domains to lead clinical diagnostics in tough circumstances. In critical care settings, AI-based technologies can also help to speed up patient diagnosis. Participants also mentioned how AI might help clinical laboratories research illness epidemiology through Big Data science, bringing insights into patients and predictive analyses. Finally, one participant presented a positive personal experience in which AI was utilised to identify a myeloma diagnosis from a highly viscous blood test that had previously been rejected, demonstrating AI’s promise in laboratory medicine.

In the discussion, a subset of participants (*n* = 3) delved into the potential benefits of AI in research and its integration with Big Data, precision medicine, and biobanks to enhance analysis and pattern recognition. According to one participant, AI algorithms possess the capability to proficiently manage and analyze large datasets, uncovering hidden patterns and correlations that may escape human researchers. This not only improves our understanding of disease mechanisms but also aids in the discovery of biomarkers and enables more accurate predictions of treatment outcomes.

**Table 1:** Potential AI application areas identified through semi-structured interviews from laboratory professionals in clinical chemistry practice

Domains
Big Data (patient information, test results, and instrument outputs) utilization for clinical service, education, and research
Budgetary planning
Decision support systems with AI algorithms using patient data and clinical guidelines
Delta checks and error detection for patient safety
Digital imaging and pattern recognition of electrophoresis gels and chromatograms
Enhance laboratory efficiency and decision making
Lean processes and workflow optimization
Managing inventory
Pattern recognition for diagnostic and prognostic assistance
Policy development (disease surveillance, epidemiology and allocation of resources based on disease burden)
Predictive models for diseases
Quality control data analysis to identify potential errors or inconsistencies
Reference range development through big data
Research on Big Data and AI (precision medicine, identification of novel biomarkers and development of biobanks)
Resources utilization through smart AI models
Total lab automation
Transforming diagnostic processes and improving patient outcomes via algorithms
Treatment response patterns with biomarkers

### Theme II: educational and curriculum adjustments

The need for tailored adjustments in Clinical Chemistry curriculum emerged. This included practical AI applications, hands-on training, and interdisciplinary collaboration, to ensure graduates are proficient in AI integration.

#### Improving clinical chemistry curriculum

Participants felt that there were gaps in the Clinical Chemistry curriculum regarding AI application. According to them, the PGME Clinical Chemistry curriculum in Pakistan currently only covers AI in a limited capacity, and there is no standardised curriculum or practical application of AI displayed to PG trainees by their lecturers. Furthermore, some participants raised worry about the absence of material on AI applications in Clinical Chemistry textbooks, as well as the difficulty in distinguishing between AI-related terminology. Participants also acknowledged a lack of awareness on how to develop interpretative diagnostic algorithms employing AI methods.

***P3:****“Theoretically it’s all there in your books but we are not applying it and we are not showing our postgraduate trainees the applications.”*

#### How to incorporate AI in clinical chemistry curriculum

Nine participants proposed gradually implementing AI education to build a cohort of early AI adopters. There was a split opinion among participants on which level of medical education should provide fundamental AI expertise. Some (P6, P7, P8, P9) believed that implementing AI adjustments into undergraduate (UGME) and postgraduate medical education (PGME) curriculum might have a substantial impact on their field. P4 proposed that including an introductory IT course in computer science at the UGME level could improve students’ performance in subjects like software operations, mobile health, and AI. This effort would allow interested students to have a strong understanding of AI’s fundamental concepts, easing their transition to this disruptive technology.

Postgraduate trainees should be competent to create and use diagnostic algorithms, according to the comments of eleven interviewees. Workplace-based assessments (WBA) can be used to test AI competencies for postgraduate trainees and trainee technologists. Continuing Professional Development (CPD) can help pathologists who are unfamiliar with AI bridge the knowledge gap. International organisations such as the International Federation of Clinical Chemistry (IFCC) and the American Association of Clinical Chemistry (AACC) can help to enhance fundamental AI knowledge and skills. During the interviews, some participants suggested that technologists’ courses be modified to integrate AI-related competencies, which would allow widespread deployment. Curricula should be adjusted at the institutional level to enable uniform dissemination of AI knowledge, and national-level efforts should be launched.

### Theme III: implementation techniques

In the realm of AI implementation in laboratories, participants discussed diverse techniques to enhance integration. Strategies encompassed robust training programs for lab staff, seamless interoperability of AI tools with existing systems, and the establishment of clear protocols for effective utilization. The theme highlights the importance of thoughtfully navigating the implementation landscape to unlock the full potential of AI in laboratory settings.

#### Critical factors for successful implementation of AI

Everyone agreed that a visionary leader with strong communication and motivational skills might persuade staff to accept AI in clinical laboratories. They stressed that laboratory managers should make sure their personnel are at ease with the move to AI.

***P6:****“Definitely our people need capacity building and training. You must address their apprehensions, find out the knowledge gaps and then only you can apply these AI tools.”*

Participants proposed that, to assuage laboratory personnel’s concerns about AI, its utility be proven and clarified. Laboratory AI-Leaders should be able to answer AI-related laboratory staff questions, therefore leaders can be the major target for AI education and training. The institute’s leadership must commit resources to AI implementation. To address the issues connected with AI adoption, open-minded and enthusiastic team members, including young scientists, are required.

***P9:*** “…*positive feedback and encouragement will help. If people can see results coming out of it themselves, I think they’ll be motivated.”*

It is critical to focus on the future of laboratory services and to cultivate advocates and project successors for AI in Clinical Chemistry. Participants emphasised how the chemical pathology community comes together on the PSCP and IFCC platforms, and how these scientific organisations have historically been supportive of introducing novel innovations to clinical laboratories.

***P7:****“Today we are a positive energetic group of chemical pathologists across Pakistan. Now we must support each other. Collegiality is very important to control conflicts and drive a project to completion.”*

One of the attendees suggested that regulatory organisations may be another driver of change for AI leaders. Few panellists discussed how accurate and trustworthy data is required for AI algorithms to perform properly. As a result, guaranteeing data quality and integrity is important to successfully applying AI in clinical laboratories.

#### Interprofessional collaboration in AI implementation

Participants agreed that AI will increasingly assist in accurate medical diagnoses. They stressed the importance of a multidisciplinary approach involving healthcare professionals, patients, and data scientists to develop algorithms integrating individual data. A participant highlighted that Clinical Chemistry faculty, considering their significant role in healthcare, are highly suitable to take the lead in developing self-learning diagnostic algorithms and utilizing Big Data to enhance healthcare outcomes and generate value.

Consensus was reached among all participants that incorporating AI advancements into laboratory practices necessitates collaboration with AI specialists from various disciplines.

According to one participant, it was recommended that the IT department be the first department to seek assistance in adapting to AI-related changes. This suggestion was because the IT department is typically responsible for managing technological infrastructure, data management, and software integration within an organization.

One participant proposed the creation of subspecialties in Clinical Chemistry as a means of training laboratory professionals in AI science, anticipating the potential impact of this disruptive innovation. Participants elaborated on the importance of global interdepartmental cooperation, especially with individuals who possess practical AI knowledge, in accelerating the broad adoption of AI on a larger scale. They further highlighted that interdepartmental collaboration can support the development of practical AI skills among Clinical Chemistry laboratory staff through the implementation of small-scale projects.

***P3:*** “*We can develop multidisciplinary working groups including people from IT, from lab’s different sections who can work on specific questions*”.

Participants explored the advantages of collaboration between pathologists and specialists from other domains to overcome limited knowledge of AI. They acknowledged that Clinical Chemists or Chemical Pathologists may face constraints due to their existing responsibilities and workload in clinical laboratories, making it impractical for them to become AI specialists independently.

***P3:****“Engaging experts form areas who have already applied this in their own fields. That would be very helpful”.*

#### Big data and AI in the future of healthcare

As many respondents discussed the future of AI in respect to Big Data, we have analyzed this aspect separately to gain a deeper understanding. From the insights gathered during our interviews with the participants, a unanimous consensus emerged - all 13 interviewees shared a common perspective regarding a significant obstacle in the realm of AI and Big Data, and that was the challenge of data curation. Concerns ranged ranging from data quality and cleanliness to data privacy and security. Some participants emphasized the need for streamlined data collection processes, while others stressed the importance of developing robust data governance frameworks. Additionally, data interoperability, integration, and ethical considerations were consistently brought into the conversation. The track record of machine learning in error detection, visionary leadership fostering innovation, the regular discourse on AI-driven approaches in international conferences, the rising demand for comprehensive data insights from clinicians, the pressing need for predictive medicine amid a high non-communicable disease burden, the goldmine of lab data marked by volume, velocity, and variety, and the proactive motivation to develop and implement AI algorithms were some of the positive forces identified. Together, these elements signify a readiness within clinical labs to lead in the utilization of AI and Big Data, promising transformative advancements in patient care and health care research. Participants understood the ethical dilemmas that needed attention and Big Data processing obstacles like data sources being inconsistent, not easily accessible, not refined, and difficult to obtain in clinical laboratories of the country.

## Discussion

This qualitative study explores the perspectives of Clinical Chemistry laboratory professionals, shedding new light on their opinions regarding the influence of AI on their practices and patient care. Over the years, AI has made significant strides in the field of medicine, leading to notable advancements in specialized areas such as enhanced clinical decision-making, improved laboratory management, and notable progress in research [19, 21]. This study contributes to the existing literature by addressing a specific and significant aspect of incorporating AI applications in Clinical Chemistry laboratories. The analysis of the interviews revealed the reasons behind participants’ hesitancy toward AI utilization, highlights the need for changes in medical education and curricula to align with the implementation of AI in clinical laboratories and it explores strategies for implementing AI in Clinical Chemistry laboratories.

The findings of the present study indicate that participants expressed approval for the transition, while also anticipating challenges and underscoring the necessity for enhancing AI-related capabilities. Concerns discussed were the lack of awareness of how AI may affect their practises, as well as the absence of AI in education and clinical laboratory practises. A poll of laboratory professionals in the United States (*n* = 1721) revealed that 72% voiced scepticism or said that they have never used AI in their usual lab work [22]. According to survey results from the United States on AI conducted on pathologists (*n* = 23), medical students (*n* = 24), physicians (*n* = 25), and radiologists (*n* = 26), 80% of participants believed AI will be useful in their practise soon [21]. Despite the availability of some previously published work, the current Clinical Chemistry and AI research landscape lacks dedicated studies that specifically study the convergence of AI, Clinical Chemistry laboratory services, and the role of medical education.

As emphasised in pertinent publications [6, 20–22], medical students and future clinicians, particularly pathologists, will need proficiency in utilising AI in their daily practise. This is because of the anticipated disruption created by technological developments that they would face during their careers. According to the current study findings, with the increasing presence of AI in the laboratory, there is a growing need to enhance education for professionals in Clinical Chemistry to adapt to this shift in practise. The necessity to update existing curricula to accommodate AI, as well as ways for incorporating AI into educational programmes, were investigated. The interview findings clearly show that AI training in pathology residency programmes is frequently insufficient in Pakistan and varies among programmes. AI education frequently falls short of the required depth, breadth, and organised approach when compared to more traditional fields of pathological instruction. Due to a dearth of faculty knowledge and available training resources in Pakistan, Clinical Chemistry residency programmes encounter difficulty in properly training residents in AI. In the United States, however, three organisations, namely the College of American Pathologists, the Association of Pathology Chairs, and the Association for Pathology Informatics, collaborated to create the “Pathology Informatics Essentials for Residents”- PIER curriculum, which is specifically designed to build proficiency in pathology informatics [18, 23]. The integration of Big Data science and AI will bring about increased efficiency, necessitate changes in laboratory infrastructure, and necessitate a shift in workforce training, requiring modifications in pathologists and technologists training curricula especially in low middle income countries [23]. At present, these solutions heavily rely on commercially available datasets primarily sourced from high-income countries, which consist of populations living near hospitals and with easier access to healthcare [24]. In contrast, lower-income countries like Pakistan face challenges in generating and refining data, often due to limited infrastructure and expertise. Consequently, a significant portion of the global population is excluded from participating in most healthcare research and innovations.

Our research gives detailed insights and contributes to the creation of strategies and educational interventions that can help pathologists in Pakistan and around the world adjust to the changes brought about by AI technologies. Addressing and combining these criteria is a task that cannot be met by a single discipline in isolation, such as pathology. A collaborative method including a team of AI experts and Clinical Chemistry pathologists for integrating AI into laboratory practises. Encouraging input and collaboration with other departments specialising in AI can improve the integration process’s efficacy [14, 22]. In another qualitative interview base study (*n* = 24), pathologists indicated that they didn’t feel the need to know every step in the algorithm’s decision-making process, as long as the outcomes could be validated and trusted [25].

Participants emphasized the significant daily data generation in their Clinical Chemistry laboratory, encompassing a wide range of information, from basic numerical results such as sodium measurements to intricate outputs from “omics” analyses, along with quality control results and vital metadata [26]. Despite laboratory medicine databases being a valuable source of data, they often lack suitability for the application of data science techniques. Certain medical difficulties can be efficiently solved using the computing power of AI solutions when the data is of high quality and supplied in the suitable manner [27]. Furthermore, the participants stressed the necessity for data standardization to facilitate the widespread adoption of AI in laboratories throughout the country. A crucial initiative for efficient Big Data utilization could be the adoption of the Findability, Accessibility, Interoperability, and Reusability (FAIR) Guiding Principles [22]. These FAIR Guiding Principles specifically aim to empower machines to automatically locate and utilize data through well-defined metadata, while also supporting its reuse by individuals within the research and clinical community. All respondents in the current study emphasized the significance of data curation as the initial step for bringing AI into laboratory settings in Pakistan. The interviewees’ responses concerning the Clinical Chemistry-AI-Big Data transformation are divided into positive and negative forces impacting the journey. Among the propelling factors are past local instances of successful AI projects, small past AI based projects, proactive leadership, the demand for diagnostic algorithms from clinicians, and the recognition of data potential. On the other hand, challenges arise from data reliability concerns, the inconsistent nature of Big Data sources in laboratory information systems, accessibility issues, inadequately refined data leading to difficulties in deriving meaningful insights, ethical quandaries necessitating attention, and obstacles in data processing. One participant used an analogy, comparing bad data to unrefined oil that holds value but requires processing to become useful. Just as oil needs refining to create valuable products like gas, plastic, and chemicals, data must be aggregated, processed, and curated for AI related solutions. Participants emphasized the importance of clinical labs implementing LOINC codes to improve data compatibility and infrastructure, thereby nurturing the national electronic health records. Wen X et al. introduced ‘Clinlabomics’, combining clinical laboratory medicine data with AI. This method extracts extensive feature data from sources like blood, fluids, and test results, enabling statistical analysis and machine learning [28].

The laboratory’s stepwise approach to AI intelligence begins with the selection and pre-processing of initial data, followed by careful consideration of the appropriate learning algorithm, and finally, thorough evaluation and validation of the obtained results [29]. Even if clinical laboratory experts are not responsible for developing AI algorithms, they can help guide the selection process by evaluating algorithms based on medical and biological data [30]. For substantial implementation to occur, lab professionals in Clinical Chemistry must eventually adopt AI tools. This acceptance will be strengthened by rigorous clinical validation, a demanding standard that often surpasses what companies are typically willing to pursue [31]. In order to avoid post-hoc interventions once the AI algorithm is available in the market, it is crucial to conduct a by-design safety assessment, overseen by expert pathologists and clinicians [32]. Laboratory specialists must reconsider their approach to patient care. They can improve workflows and devote more time and resources to enhancing patient care by applying AI into test selection and interpretation [5]. Lippi and Plebani advocate for a new future in Clinical Chemistry and laboratory medicine, criticizing the current practice of simply relaying laboratory results without context. Instead, pathologists should use their expertise to interpret results and provide valuable clinical information, taking on the role of ‘diagnostic stewards’ [33]. AI integration in pathology needs customised training programmes that equip pathologists to use AI technologies efficiently, optimise diagnostic accuracy, and improve patient care. To ensure that clinicians understand their role as ‘diagnostic stewards employing AI tools,’ each professional in the Clinical Chemistry laboratory must describe their purpose within the hospital setting.

The current study has some limitations that should be acknowledged. Firstly, there is an imbalance in the distribution of participants, with a majority being academics or practicing chemical pathologists from private institutes in Pakistan. Nonetheless, the study manages to encompass a diverse spectrum of volunteers, each possessing varying degrees of experience within clinical laboratories. This participant pool encompasses a broad array of experts, ranging from seasoned pathologists to those currently in training, as well as individuals with differing levels of involvement in the development of AI, both active and passive. It is important to note that the participants may not fully represent the entire population of pathologists in Pakistan who are undergoing training or working in clinical laboratories. Since the study exclusively involves participants from Pakistan, the findings may have limited generalizability to other countries with different healthcare systems, cultural backgrounds, or levels of technological usage. The authors acknowledge the rapidly evolving nature of AI and hence the study findings may be time sensitive. Despite these limitations, this research provides a strong foundation for future investigations and highlights existing gaps guiding the way for future investigations in this dynamic field of AI.

## Conclusions

According to the study’s findings, individuals working in clinical laboratories in Pakistan, regardless of their career and experience levels, demonstrate limited understanding of AI in Clinical Chemistry practices. It is crucial to establish a foundational understanding of AI to effectively integrate AI-related advancements into standard laboratory practices. However, the lack of AI curricula, skills, and functioning AI models in current laboratory practices complicates the process of implementing change. The study’s outcomes provide a solid framework for offering recommendations to clinical laboratories, scientific bodies, and national and international Clinical Chemistry and laboratory medicine societies on managing the evolving practices of pathologists due to AI.

## Data Availability

The datasets generated and/or analysed during the current study are not publicly available as most of the information gathered for this study is presented as quotations and qualitative analysis but are available from the corresponding author on reasonable request.
